# Prediction of conformationally dependent atomic multipole moments in carbohydrates

**DOI:** 10.1002/jcc.24215

**Published:** 2015-11-08

**Authors:** Salvatore Cardamone, Paul L. A. Popelier

**Affiliations:** ^1^ Manchester Institute of Biotechnology (MIB)131 Princess StreetManchesterM1 7DNGreat Britain; ^2^ School of ChemistryUniversity of ManchesterOxford RoadManchesterM13 9PLGreat Britain

**Keywords:** quantum theory of atoms in molecules, carbohydrates, quantum chemical topology, conformational sampling, kriging, electrostatics, multipole moments

## Abstract

The conformational flexibility of carbohydrates is challenging within the field of computational chemistry. This flexibility causes the electron density to change, which leads to fluctuating atomic multipole moments. Quantum Chemical Topology (QCT) allows for the partitioning of an “atom in a molecule,” thus localizing electron density to finite atomic domains, which permits the unambiguous evaluation of atomic multipole moments. By selecting an ensemble of physically realistic conformers of a chemical system, one evaluates the various multipole moments at defined points in configuration space. The subsequent implementation of the machine learning method kriging delivers the evaluation of an analytical function, which smoothly interpolates between these points. This allows for the prediction of atomic multipole moments at new points in conformational space, not trained for but within prediction range.

In this work, we demonstrate that the carbohydrates erythrose and threose are amenable to the above methodology. We investigate how kriging models respond when the training ensemble incorporating multiple energy minima and their environment in conformational space. Additionally, we evaluate the gains in predictive capacity of our models as the size of the training ensemble increases. We believe this approach to be entirely novel within the field of carbohydrates. For a modest training set size of 600, more than 90% of the external test configurations have an error in the total (predicted) electrostatic energy (relative to *ab initio*) of maximum 1 kJ mol^−1^ for open chains and just over 90% an error of maximum 4 kJ mol^−1^ for rings. © 2015 Wiley Periodicals, Inc.

## Introduction

The computational analysis of biochemical systems is largely biased toward peptides and proteins. One may, therefore, be forgiven for assuming that they possess a near monopoly in biochemistry. However, it is only by complexation with additional molecular species that peptides and proteins are able to accomplish their myriad roles within biological systems.^1^ For example, eukaryotic proteins are subject to post‐translational modification, a process in which various carbohydrate sequences are attached to the protein. Vital chemical entities, such as enzymatic cofactors (e.g. ATP, NADP, etc.) and nucleotides, are entirely dependent upon the existence of products from the pentose phosphate pathway, which are synthesised from carbohydrates. In fact, the phosphate pathway would be unable to run at all if it were not for the energy derived from carbohydrates, which undergo glycolysis and are subsequently passed into the tricarboxcylic acid cycle.

Many biochemical force fields are parameterised by exhaustively sampling quantities arising from peptide atom types. One cannot simply use protein atom types as a direct substitution for carbohydrate atom types for a number of reasons:

1. Peptides possess features that are generally absent in carbohydrates, most prominently the presence of nitrogen and the ability to form structural motifs. Both of these features are particularly perturbative to electrostatic quantities associated with constituent atoms. For example, nitrogen possesses a significant quadrupole moment, which influences other atomic electrostatic quantities anisotropically, and cannot be captured by the standard point charge approximation. Equivalently, structural motifs such as helices and sheets are stabilised by vast intermolecular bonding networks. This dependence on structural motifs necessarily influences the properties of other atoms by constraining them to states that do not necessarily coincide with those of the unfolded non‐native state. Although carbohydrates do form structural motifs, they tend to remain flexible under standard biological conditions, and do not typically assemble into the stable secondary structures, which polypeptides do.
2. Carbohydrates exhibit much more conformational freedom than peptides. Electronic quantities vary as a function of the conformational degrees of freedom of a molecular species^2^. As such, the electronic quantities of a more flexible conformation will vary to a greater extent than those of a less flexible one.
3. Many carbohydrate species exhibit a preference for axial rather than equatorial arrangements of electron‐rich substituents on an anomeric carbon. The origin of this anomeric effect is not entirely clear,^3^ but the energetics that arise from it must be captured by a force field which deals with carbohydrates. Similarly, the exo‐anomeric effect, which deals with substituents linked to an anomeric oxygen, forces a separate conformational preference. It is not within the scope of this work to deal with the anomeric and exo‐anomeric effects in any great detail, and so we refer the interested reader to a more rigorous overview.^4^
4. Similar to the anomeric effect, the gauche effect can arise within a number of carbohydrate species and bias rotamer preferences.^5^ This effect has an ambiguous origin. Research has suggested it to arise from hyperconjugation or solvent effects. In short, it is the preference of a gauche rotamer over an antirotamer, where the latter would be stereoelectronically preferable. Work by Kirschner and Woods^[6]^ proposed that the gauche effect results from solvent effects, which are not important in our work since we are explicitly dealing with gas phase molecules. However, for a carbohydrate force field to be of use, this effect must be accounted for.

The conformational freedom of carbohydrates renders them somewhat troublesome for experimentalists, as they prove to be highly difficult to characterize by conventional high‐resolution structural determination techniques,^7^ particularly X‐ray crystallography. To be precise, carbohydrates tend to be difficult to crystallize, which is problematic because X‐ray diffraction techniques are a valuable source of structural information. As such, the structural characterization of carbohydrates rests with a few experimental techniques, and subsequent validation by computational means. This required harmony between experiment and computation is vastly important, and has been recently explored,^8^, ^9^ and so proves to be a fruitful avenue for development.

Classical force fields such as OPLS‐AA, CHARMm, GROMOS, and AMBER appear to have characterized carbohydrates as “secondary molecular species” relative to their peptide counterparts. As such, these parameterizations resemble “bolt‐on” components. However, force fields that are specifically tailored for carbohydrates do exist and have proven successful. GLYCAM^10^ is perhaps the most prominent of these force fields, and has been ported to AMBER. More recently, the advent of DL_FIELD has facilitated the use of GLYCAM parameters within DL_POLY 4.0.^11^ GLYCAM has undergone extensive validation in an attempt to demonstrate its efficacy. Several studies have focused upon its ability to reproduce conformer populations in explicitly solvated molecular dynamics (MD) simulations,^6^, ^12^, ^13^ which is obviously important owing to the massive conformational freedom of carbohydrates. The applicability of GLYCAM to larger, more biologically relevant structures, such as the binding of endotoxin to recognition proteins^14^ or the dynamics of lipid bilayers,^15^ has also been demonstrated.

GLYCAM has attempted to break the paradigm of deriving partial charges based on a single molecular configuration. Instead, it has been developed such that the partial charges are averaged over the course of a MD simulation, thus (albeit simplistically) accounting for the dynamic nature of electronic properties.^16^ However, it must be emphasised that GLYCAM resides within the partial charge approximation to electrostatics, thus severely limiting its predictive capacity. Sugars are particularly amenable to hydration, yet a partial charge approximation to electrostatics cannot recover the directional preferences of hydrogen bond formation without the addition of extra point charges at non‐nuclear positions. The isotropic nature of partial charge electrostatics is readily overcome by use of a multipole moment description of electrostatics, which naturally describes anisotropic electronic features such as lone pairs. The benefits of such a multipole moment description over their partial charge equivalents has been systematically demonstrated over the past 20 years in many dozens of papers, recently reviewed.^17^ These benefits are not necessarily outweighed by the common misconception that multipole moment implementations are computationally expensive relative to their point charge counterparts. The long‐range nature of point charge electrostatics, 𝒪(r^−1^), relative to higher order multipole moments [dipole‐dipole interactions, for example, die off as 𝒪(r^−3^)], means this is not strictly true. Point charges require a larger interaction cutoff radius relative to higher order multipole moments, and, therefore, form the bottleneck in electrostatic energy evaluation. Given proper handling (e.g. parallel implementation), the computational overheads associated with multipole moment electrostatics can be managed.

In the remainder of this article, we shall demonstrate a novel means for modelling electrostatics by use of a multipole moment expansion centred upon each atomic nucleus. The techniques we present will inherently capture the conformational dependence of these multipole moments.

## Methodology

### Atomic partitioning

The development of molecular orbital theory largely caused a decline in chemical understanding of the theoretical description of molecular systems. The fact that each electron occupies a molecular orbital dispersed across the spatial extent of the molecule gave rise to a valid query: why do functional groups impart some property, such as reactivity, to the molecule, when the distribution of electrons throughout the system is essentially no different to the inert species? Surely, there must be some localization of electrons to the functional group, which permits subsequent functionality. If this is not the case, then even the most fundamental chemical concepts, such as those of nucleophiles and electrophiles, have no theoretical grounding. These terms are used to denote a property of an atom in a molecule, which is not recovered by molecular orbital theory. For example, if one assesses butanol by means of molecular orbital theory, the electrons “belonging” to the hydroxyl group are dispersed throughout the entirety of the molecule. If this is truly the case, then it becomes particularly problematic when one attempts to explain why the presence of the functional group imparts reactivity (an electronic phenomenon), when the electrons are not localized.

Bader and co‐workers^18^ went some way to address this problem by performing an appealing partitioning of three‐dimensional space into atomic basins, which pictorially defines an “atom in a molecule,” an approach called the Quantum Theory of Atoms in Molecules (QTAIM). The latter is the first segment of a broader approach called Quantum Chemical Topology (QCT),^19^, ^20^, ^21^, ^22^ which analyzes quantum mechanical functions other than the electron density and its Laplacian. The QTAIM partitioning has been demonstrated to have several advantages compared to other partitioning schemes,^23^, ^24^, ^25^ and enjoy excellent transferability compared to other schemes.^26^ By use of Born's interpretation of quantum mechanics, one generates a physical electron density, $\rho \left(r\right)$, from an *ab initio* wavefunction, $\psi \left(r\right)$, obtained entirely from a first principle calculation. This electron density is then be completely partitioned by use of the gradient operator

(1) $$\nabla \;=\frac{\partial }{\partial x}\hat{i}\;+\;\frac{\partial }{\partial y}\hat{j}\;+\;\frac{\partial }{\partial z}\hat{k}$$

where $\hat{i},\;\hat{j},\;\hat{k}$ are unit vectors along the *x, y,* and *z* axes, respectively, to generate the vector $\nabla \rho \left(r\right)$. The evaluation of the gradient of a scalar field results in a vector field, the vectors of which are directed along the path of greatest increase in a function. As such, the vectors that define the field $\nabla \rho \left(r\right)$ point toward the greatest increase in the scalar field $\rho \left(r\right)$. If one were to map $\rho \left(r\right)$ by use of a contour plot, such that each contour represented the encasing of a surface with constant electron density, termed an isosurface, then each vector in $\nabla \rho \left(r\right)$ would intersect each isosurface orthogonally.^27^

Points in the vector field defined such that $\nabla \rho \left(r\right)=0$ are termed critical points. Within the scalar field $\rho \left(r\right)$ they represent a maximum, minimum or saddle point (mixture of minimum or maximum depending on direction). The identity of each critical point is revealed by assessing the curvature of $\rho \left(r\right)$ at each point, achieved by evaluation of the Hessian of $\rho \left(r\right)$

(2) $$H\left(\rho \right)\;=\;\left[\begin{array}{ccc} \frac{\partial^{2}\rho }{\partial x^{2}} & \frac{\partial^{2}\rho }{\partial x\partial y} & \frac{\partial^{2}\rho }{\partial x\partial z}\\ \frac{\partial^{2}\rho }{\partial y\partial x} & \frac{\partial^{2}\rho }{\partial y^{2}} & \frac{\partial^{2}\rho }{\partial y\partial z}\\ \frac{\partial^{2}\rho }{\partial z\partial x} & \frac{\partial^{2}\rho }{\partial z\partial y} & \frac{\partial^{2}\rho }{\partial z^{2}} \end{array}\right]$$

This Hessian matrix is a real symmetric matrix and hence Hermitian. Therefore, its eigenvalues are real and they express the magnitude of the curvature along each of the principal axes, which are marked by the direction of the corresponding eigenvectors. The nature of the critical point in question is then given by two easily evaluated parameters: the rank ( ω) and signature ( σ) of the critical point, where the former is defined as the number of nonzero eigenvalues of $\rho \left(r\right)$, and the latter as the sum of the signs of the eigenvalues.

A fundamental result in the topology of $\nabla \rho \left(r\right)$, of great importance in the following, is the partitioning of a molecular system into topological atoms. A key feature necessary to achieve this result is the gradient path. An easy way to grasp what this is to think of a succession of very short gradient vectors, one after the other and constantly changing direction. In the limit of infinitesimally short gradient vectors, one obtains a smooth and (in general) curved path, which is the gradient path. A gradient path always originates at a critical point and terminates at another critical point. Bundles of gradient paths form a topological object depending on the signature of the critical points that the object connects. All possibilities have been exhaustively discussed before^28^ but three ubiquitous possibilities are specified as follows: (i) the topological atom is a bundle of gradient paths originating at infinity and terminating at the nucleus, (ii) the bond path (or more generally atomic interaction line) is the set of two gradient paths, each originating at a bond critical point and terminating at a different nucleus, and (iii) the interatomic surface (IAS), which is a bundle of gradient paths originating at infinity and terminating at a bond critical point.

An interatomic surface obeys the following condition

**Figure 1:**
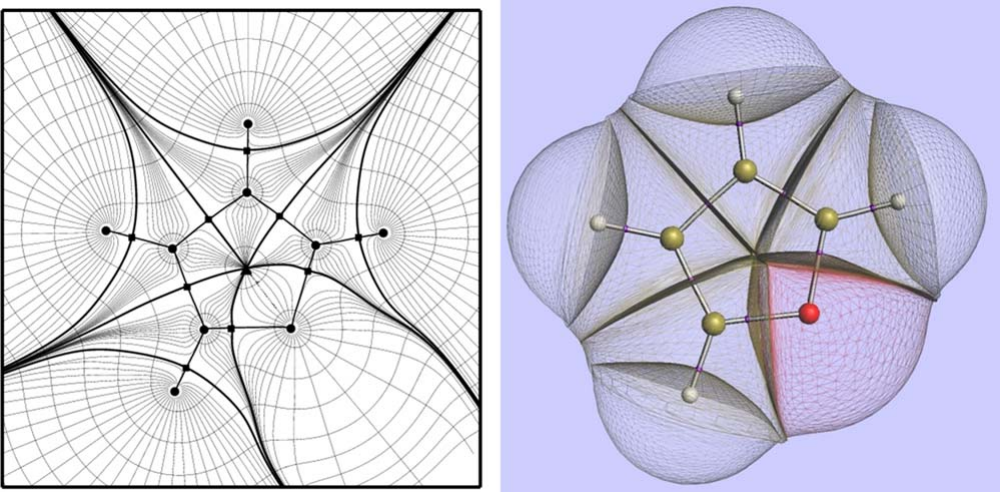
(left) A contour plot of the electron density of in molecular plane of furan superimposed onto a representative collection of gradient paths. Atoms are represented by black circles, where the gradient paths terminate. Interatomic surfaces are highlighted as solid curves, and contain bond critical points (black squares). A ring critical point (triangle) is also shown in the center of the furan. (right) A 3D representation of the topological atoms in furan, in the same orientation as the left panel. The molecule is capped by the ρ = 0.0001 au envelope and the bond critical points are marked in purple.

(3) ∇ρ(r)•n(r)=0 ∀r∈IAS

where $n\left(r\right)$ is defined as the vector normal to the IAS. By finding all surfaces that obey this condition, the molecule is completely partitioned into topological atoms $\Omega_{i}$, where the subscript denotes the atomic basin associated with the $i^{th}$ atom in a molecule. All key topological features of $\nabla \rho \left(r\right)$ are summarized in Figure 1.

Integration over these atomic basins allows atomic properties P_f_(Ω) to be defined and calculated. The universal formula from which all atomic properties can be calculated is

(4) $$P_{f}(\Omega )=\int_{\Omega }d\tau \;f(r)$$

where integration with respect to dτ denotes a triple integration over all three Cartesian coordinates, confined to the atomic volume Ω, and f(**r**) denotes a property density. For example, if f(**r**) equals the electron density ρ(**r**) then the corresponding atomic property is the electronic population of the topological atom. If *f*(**r**) = 1, then we obtain the atomic volume and when *f*(**r**) = ρ(**r**)R_ℓm_(**r**) the topological atom's multipole moments,^29^ where R_ℓm_(**r**) is a spherical tensor^30^ of rank ℓ and *m*. Others have shown^31^ the better agreement with reference electrostatic potentials of topological multipole moments compared to CHELPG charges. A further advantage of QCT is that the finite size and nonoverlapping nature of the topological atoms avoids the penetration effect, which may otherwise appear in the calculation of intermolecular interaction energies.

### Kriging

We can only outline kriging here, for more technical details the reader is referred to our work on histidine.^32^ In general, a machine learning method is trained to find a mapping between an input and an output. The machine learning method kriging^33^, ^34^, ^35^ can also be seen an interpolative technique able to predict the value of a function at an arbitrary d‐dimensional point, $x^{*}$, given the value of the function at n different points, $\left\{x_{1},x_{2},\ldots ,\;x_{n}\right\}$, in this d‐dimensional space. Kriging is apt at modeling high‐dimensional function spaces, and so is particularly stable when considering the conformational space of large molecules. A cornerstone of kriging is that if two input points are very close together in space then their output values are also very close. To put this more formally, consider the example of three points $x_{\;}$, $x^{\prime },$ and $x^{*}$ such that $x^{*}$ is closer to $x^{\;}$ than to $x^{\prime }$ within the d‐dimensional space, i.e. $\left|x^{*}-x^{\;}\right|<\left|x^{*}-\;x'\right|$. As a result, the function values of $f\left(x\right)$ and $f\left(x^{*}\right)$ should be correlated more so than the values of $f\left(x^{\prime }\right)$ and $f\left(x^{*}\right)$.

The function values $\left\{f\left(x_{1}\right),\;f\left(x_{2}\right),\ldots ,f\left(x_{n}\right)\right\}$ from which $f\left(x^{*}\right)$ is composed form a basis set that is necessarily only complete if each point within d‐dimensional space has been sampled, that is, as n→∞. Hence, kriging only gives an approximate value for any predicted point $f\left(x^{*}\right)$, and the accuracy of its prediction increases as n→∞. An adequate kriging model typically requires at least 10 × d data points, which is well within reach of modern day computational power for high‐dimensional functions, and so kriging is a feasible machine learning method for our purposes.

**Figure 2:**
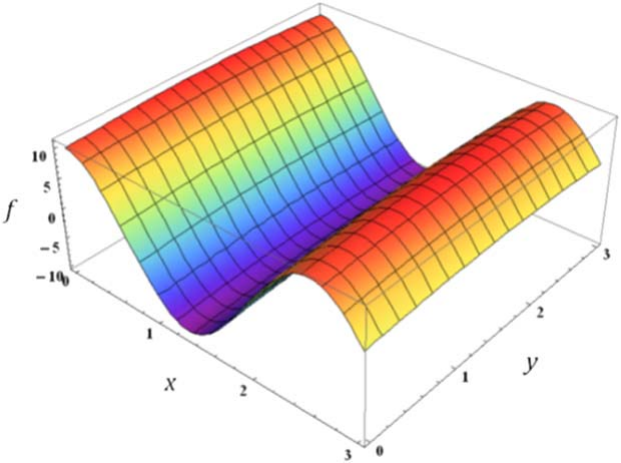
Plot of $f\left(x,y\right)$ against the two dependent variables. Note how the value of $f\left(x,y\right)$ is relatively invariant in *y* compared to *x*, which results in $\theta_{x}>\theta_{y}$.

There are several intricacies involved with the evaluation of a kriging model, in particular the manner by which multidimensional functions are dealt with. For a d‐dimensional vector in function space, x, each dimension, or *feature*, is assigned a parameter $\theta_{d}$, which maps the variance of $f\left(x\right)$ with respect to a change in the $d^{th}$ feature. Consider, for example, the function mapped in Figure 2. In relative terms, whilst $f\left(x,y\right)$ changes significantly in response to a change in x, it is essentially invariant with respect to a change in y. As such, y is relatively insignificant when assessing the correlation between two points in function space, i.e. $f\left(x,y\right)$ is much more dependent on x than on y. As a result, x is assigned a higher “importance” than y, which is reflected in the value of $\theta_{x}$, where $\theta_{x}>\theta_{y}$.

Kriging is a kernel method in view of the kernel function at its heart. This function enables kriging to operate in a high‐dimensional, *implicit* feature space without ever computing the coordinates of the data in that space. Instead, only the inner products between the images of all data pairs in feature space need to be computed. The kernel that we evaluate when obtaining a kriging model is a function of the correlation between two points in feature space, $x^{i}$ and $x^{j}$, such that the correlation between the points, $R\left(x^{i},x^{j}\right)$, is given by

(5) $$R\left(x^{i},x^{j}\right)=\exp\left[-\sum_{h=1}^{d}\theta_{h}{\left|x_{h}^{i}-\;x_{h}^{j}\right|}^{p_{h}}\;\right]$$

Brief analysis of this function shows that, if $x_{h}^{i}$ and $x_{h}^{j}$ are situated closely together for many features *h*, then the argument of the exponential tends toward zero, leading the correlation between the two points to tend toward one. Note that if the *h^th^* feature is relatively unimportant, it will be assigned a low $\theta_{h}$ value. As a result, the *h^th^* term in the sum becomes smaller if $x_{h}^{i}$ and $x_{h}^{j}$ are relatively far apart, leading to an increased correlation between the two points, which demonstrates that $f\left(x_{h}^{i}\right)$ and $f(x_{h}^{j})$ are similar.

Given a set of n points in feature space, an n×n correlation matrix, ${\hat{R}}_{\;}$, is defined, whose elements are defined as the correlation between the $i^{th}$ and $j^{th}$ points (and as such is symmetric). The task at hand is then to minimize the mean squared error of prediction of the kriging estimator. It can be shown that this is equivalent to maximizing the likelihood function *L*, which is given by

(6) $$L=\;\frac{1}{{\left(2\pi \right)}^{\frac{n}{2}}{\left(\sigma^{2}\right)}^{\frac{n}{2}}{\left|R\right|}^{\frac{1}{2}}}exp\left[\frac{-{\left(y-1\mu \right)}^{t}R^{-1}\left(y-1\mu \right)}{{2\sigma }^{2}}\right],$$

where **1** is a column vector of ones, *t* denotes the transpose, $\sigma^{2}$ is the process variance, and μ is a constant term that models the global trend (i.e. “background”) of the column vector **y** of observations. This formula arises from the definition of a Gaussian process and is not discussed here. For our purposes, it is more convenient to maximize the natural logarithm of this function, which is done analytically (see Supporting Information of Ref. 36) by differentiation with respect to σ^2^ and μ and setting the respective derivatives to zero. When these optimal values for σ^2^ and μ are substituted back into eq. (6), one obtains the “concentrated” log‐likelihood function^32^, or

(7) $$\log L=\;-\frac{n}{2}\log\left({\hat{\sigma }}^{2}\right)-\frac{1}{2}\log\left(\left|\hat{R}\right|\right)$$

where L is the likelihood and $\hat{\sigma }$ is the process variance (a constant). The parameters θ and p, which are *d*‐dimensional vectors containing the individual feature parameters mentioned previously, must be optimized, which is equivalent to maximizing the “concentrated” likelihood function. From eqs. (5) and (7) it is clear that *log L* is a function of θ and p. The function *log L* is the quantity that needs to be maximized, which is done by another machine learning method called particle swarm optimization.^37^ We can then make a prediction of the output at a new point $x^{*}$ with the optimized kriging parameters θ and p, using the formula

(8) $$\hat{y}\left(x^{*}\right)=\;\hat{\mu }+\sum_{i=1}^{n}a_{i}\cdot \varphi \left(x^{*}-x^{i}\right),$$

where $a_{i}$ is the *i^th^* element of the vector $a=R^{-1}(y-1\hat{\mu })$ where $\hat{\mu }$ is the (known) maximized mean μ, while $\varphi \left(x^{*}-x^{i}\right)$ is calculated^[32]^ via eq. (5).

In our implementation of machine learning, each atom within a molecule is termed a *kriging center*, with a respective multipole expansion (up to the hexadecapole moment) centered on the nucleus. Higher rank multipole moments are highly sensitive to the change in conformation of the molecule due to a fluctuating electric field. As such, we define each multipole moment as a function of the 3N‐6 degrees of freedom of the molecular system, pertaining to each kriging center.

The molecule is distorted by means of energy input into each of its normal modes (discussed in section 2.3), and the multipole moments of each kriging center elucidated for a given training set size. These data are used to construct separate kriging models for each kriging center. From this, the kriging model is then able to, given an arbitrary point in conformational space, predict the associated multipole moments which accompany such a position. This method has been developed and tested substantially within our group, and gives very agreeable results for a number of distinct chemical species,^32^, ^38^ but is nevertheless necessarily a subject of intense ongoing refinement.

### Conformational sampling

Here, we present a conformational sampling methodology that utilizes the normal modes of a molecular system as a means for dynamically evolving the system. This methodology has been used before in our lab for amino acids^32^, ^39^, ^40^ and small molecules^[41]^ but this is the first time we report it in great detail. Each normal mode has a corresponding frequency that is calculated by diagonalization of the mass‐weighted Hessian, **H**, the details of which are incorporated in the Supporting Information. With these frequencies, a system of equations of motion is obtained, which permits for the conformational evolution of the molecular system in time. These equations take a harmonic form, and are elaborated upon in the Supporting Information.

Expressing the system in a basis of internal coordinates results in six of the 3N Cartesian degrees of freedom possessing a frequency of zero (these correspond to the global translational and rotational degrees of freedom), and so we need only evaluate the *3N–6* “vibrational” equations of motion.^42^ We refer to these $N_{vib}=3N-6$ degrees of freedom in the internal coordinate basis as “modes” of motion. The transformation from a mass‐weighted cartesian coordinates, q, to the set of internal coordinates, s, is attained by evaluating the *3N x 3N* transformation matrix, 𝒟, which satisfies

(9) s=𝒟q

Note that this transformation retains the mass‐weighting of q. Construction of 𝒟 is undertaken by defining six orthogonal vectors corresponding to the global translational and rotational degrees of freedom of the system, as defined by the Sayvetz conditions. To implement these, the system must be specified in a global reference frame (sometimes termed the Eckart frame), the origin and axes of which coincide with the centre of mass and principal axes of inertia, respectively. Suffice to say that these conditions dictate the system possesses no net angular momentum relative to the Eckart frame, which rotates with the system.

The above leads to the generation a set of six orthogonal vectors, which are invariant under global translational and rotational motion. These vectors correspond to the first six columns of 𝒟. Since the internal coordinates form a mutually orthogonal basis, the resultant $N_{vib}-6$ columns are generated by means of a Gram‐Schmidt orthonormalization procedure, whereby the projection of ${𝒟}_{j}$ on ${𝒟}_{i}$, $P_{ij}$, is given by

(10) $$P_{ij}=\;\frac{{𝒟}_{i}\cdot {𝒟}_{j}}{{𝒟}_{i}\cdot {𝒟}_{i}}$$

Note that the columns of 𝒟 are also normalized by this process. If ${𝒟}_{i}$ and ${𝒟}_{j}$ are orthogonal, then $P_{ij}=0$. If $P_{ij}\neq 0$, then $P_{ij}$ is subtracted from $P_{j}$ and the process iterated until $P_{ij}=0$. Generalizing to account for our $N_{vib}$ columns,

(11) $$\begin{array}{c} D_{7}=\;D_{7}-1\sum_{i=1}^{6}P_{i7}\\ D_{8}=\;D_{8}-1\sum_{i=1}^{7}P_{i8}\\ \vdots \\ D_{n}=\;D_{n}-1\sum_{i=1}^{n-1}P_{in} \end{array}$$

where **1** is column vector of ones. As before, this procedure is iterated until $P_{ij}=0\;\forall \;i,j$, which results in the $\left\{{𝒟}_{n}\right\}$ forming a mutually orthonormal set. In computational terms, the threshold value of the $P_{ij}$, which we require before considering the $\left\{{𝒟}_{n}\right\}$ to be mutually orthonormal, is $O\left(10^{-8}\right)$.

The mass‐weighted Hessian H, outlined in Supporting Information, is transformed into the internal coordinate basis, by use of 𝒟

(12) $$H_{s}=\;{𝒟}^{⊤}H_{q}𝒟$$

where the subscripts denote the basis in which these quantities are expressed, and ^T^ denotes the transpose. To evaluate the frequencies of the various modes of motion, we require diagonalization of $H_{s}$,

(13) $${ℰ}^{-1}H_{s}ℰ=I\lambda$$

where ℰ denote the eigenvectors of $H_{s}$ and I is the identity matrix. In our protocol, this is achieved by tridiagonalizing the Hessian by the Householder algorithm, followed by a QR decomposition of the tridiagonal Hessian,^43^ yielding a diagonal Hessian, as required. The resultant eigenvalues, ${\left(I\lambda \right)}_{ii}=\lambda_{i}$, are related to the mode frequencies, $\nu_{i}$, by

(14) $$\nu_{i}=\sqrt{\frac{\lambda_{i}}{4\pi^{2}c^{2}}}\;\forall i=1,\ldots ,3N$$

where *c* is a factor which incorporates the speed of light, c, and the conversion from atomic units to reciprocal centimeters.

Of course, six of these frequencies correspond to the global translational and rotational degrees of freedom of the system and are zero, thus yielding $N_{vib}$ non‐zero frequencies. The reduced masses and force constants corresponding to the modes with ω≠0 are given by similar manipulations of these quantities. The reader is again directed to Ref. [42] for a discussion of their calculation. The amplitude of the $i^{th}$ mode, $A_{i}$, is given by rearrangement of the familiar expression for the energy of a simple harmonic oscillator, $E=k_{i}{A_{i}}^{2}/2$

(15) $$A_{i}=\sqrt{\frac{2E}{k_{i}}}$$

where $k_{i}$ is the force constant of the mode of motion, and E is the energy available to it. We now have all quantities required to evolve the modes of motion and replicate the vibrational dynamics of the system. The total energy available to the system is given by the expression for thermal energy, $E=N_{vib}kT/2$, and is stochastically distributed throughout the modes. The phase factors of the modes, ϕ, are also randomly assigned: if ϕ=0 for all modes, then they oscillate in unison, which corresponds to a photonic single frequency excitation. Instead, we assume the modes to resonate out of phase with one another, as energy transfer to each mode from an external heat bath will be predominantly decoherent.

The sole remaining issue is the choice of a dynamical timestep with which to evolve the various modes of motion. Our choice is based on the desire to ensure a single oscillation of a mode is sampled uniformly, i.e. we do not want to bias our sampling toward specific regions of the period function that describes the evolution of the mode. We obtain the time period of the mode as $T_{i}=1/\nu_{i}$, and subsequently ensure that the sampling methodology permits $n_{cycle}$ points to be evaluated along a single oscillation. In this case, the dynamical timestep for the $i^{th}$ mode, $\Delta t_{i}=1/\nu_{i}n_{cycle}$. $n_{cycle}$ is left as a user‐defined input, and is set to $n_{cycle}$ = 10 in the following work. Additionally, the distribution of the total energy throughout the modes is considered a dynamic quantity, and so for every $n_{reset}$ samples that are output, the energy is randomly redistributed throughout the system. The phase factors are also redefined at the same frequency. Again, $n_{reset}$ is left as a user‐defined parameter, and is set as $n_{reset}$ = 2 in the following.

We wish to clarify an issue in order to avoid misinterpretation. The above methodology is not meant as an exact technique for the exploration of the molecular potential energy surface (PES). By truncating the Taylor series of the potential energy at second order, we essentially model the local PES as a harmonic well, which is obviously a simplification. However, we believe the above process to be a computationally efficient means for generating molecular conformers. Moreover, the important alternative method of MD to generate conformers is not necessarily more realistic. Whilst the success of MD is not in question, the validity of the forces fields that are currently implemented is not guaranteed.

## Computational Details

The workflow proposed below essentially takes an ensemble of configurations as input, and outputs kriging models for the variation in the atomic multipole moments as a function of the configuration of the system:

1. The test system is sampled in accordance with the methods outlined in section 2.3. The general idea is to sample as much of configuration space as would form an ensemble for the true physical system along to the course of a dynamical trajectory. This subsequently allows for the formation of a kriging model that will be used in a purely interpolative context.
2. Single‐point calculations are performed on each sample and the resultant *ab initio* electron density is partitioned by QCT software. The multipole moments of the topological atoms are subsequently obtained. This allows a kriging model to evaluate a functional form corresponding to the evolution of the various multipole moments as a function of conformation.
3. The sample set is split into a nonoverlapping *training set* and *test set*. The training set is utilised for training of our kriging models, i.e. these are the points that the kriging function must pass through. The test set is not trained for, but is used after the construction of the kriging models to evaluate the errors associated with their predictions.
4. A kriging model is built for each multipole moment of each atom, which allows for the generation of a smooth interpolative function, mapping the evolution of the multipole moment against the conformational parameters of choice. By use of particle swarm optimization, we optimize our kriging parameters, $\left\{\theta ,p\right\}$ to obtain an optimal kriging model.
5. The kriging models are assessed by making them predict the multipole moments for each atom in a system whose configuration has not been used for training of the kriging model. However, we do possess the *ab initio* multipole moments for this configuration. As such, we evaluate the energy associated with all 1–5 (i.e. two nuclei separated by four bonds) and higher (1–*n*, *n* > 5) order interatomic electrostatic interactions as given by the predicted multipole moments from the kriging models, and the equivalent energy as given by the (exact or original) *ab initio* atomic multipole moments. We subsequently assess the deviation of the kriging predictions from the *ab initio* electrostatic energies.

The choice of 1–*n* (*n* ≥ 5) interactions over the conventional 1–*n* (*n* ≥ 4) serves a twofold purpose: (i) avoiding any potential divergence in the electrostatic energy between two atom‐centered multipole moment expansions, (ii) avoiding any issues from a coupling of torsional and electrostatic energetics. In other work from this lab, to be published soon, we show that short‐range electrostatic energy 1–*n* (*n* < 5) can be satisfactorily kriged. Nonelectrostatic energy contributions can be calculated within the QCT context and again adequately kriged, a result that will be published elsewhere.

Note that the electrostatic energy^[38]^ is the final arbiter in the validation of the kriging models, rather than the atomic multipole moments themselves, which are the kriging observations. The molecular electrostatic energy is calculated by a well‐known multipolar expansion^[30]^ involving a multitude of high‐rank atomic multipole moments.^41^ This expansion is truncated to quadrupole‐quadrupole (*L* = 5) and rank‐equivalent combinations (dipole‐octopole and monopole‐hexadecapole). Second, the interatomic contributions to the total molecular electrostatic energy are limited to 1–5 and higher.

Whilst somewhat indirect, the validation through energy rather than multipole moment has a twofold purpose. Primarily, the energy is the quantity that will be used for dynamical simulations, and so is the ultimate descriptor that we wish to evaluate correctly. Second, the alternative would be to assess the predictive capacity of each individual kriging model. For a system with any sizeable number of atoms, where each atom has 25 individual multipole moment kriging models, the data analysis obviously becomes overwhelming. However, this analysis is unnecessary owing to the uniqueness of the Taylor expansion from which the multipole moments arise. Since the electrostatic energy is computed from two such unique series, then if the electrostatic energy is correctly predicted, the multipole moments must also be correct by deduction. Note that this consideration is valid for a *single* atom‐atom interaction.

In order to gauge the models’ validity, we plot a graph colloquially termed an “S‐curve” owing to its typical sigmoidal shape but of course it is really a cumulative distribution function. The S‐curve plots the absolute deviation of the predicted energy from the *ab initio* energy, predicted from the *ab initio* multipole moments, after having evaluated the multipole moment interactions. Put more precisely, the predicted multipole moments form an energy that is subtracted from an energy obtained from the *ab initio* moments. Then the absolute value of this difference is taken. Hence compensation of errors is not allowed because first the difference is taken and then the absolute value. These energetic deviations are plotted against the percentile of test configurations that fall on or below the given energetic deviation.

Our aim is then twofold: the first is to reduce the upper tail of the sigmoid such that the 100th percentile error is convergent at as low an error as possible. This corresponds to the predictions being uniformly good across the test set with no spurious predicted interactions. Our second aim is to shift the S‐curve as far down the abscissa (i.e. to the left) as possible, which ensures the average error associated with our predictions is as low as possible. The first goal is achieved by certifying that the training points used for the construction of the kriging models form the boundaries of configurational space with respect to our sample set. This boundary checking guarantees that the kriging model is being asked to interpolate from training data. Boundary checking has not yet been implemented, but we propose a simple means by which this could be accomplished. The initial geometry from which we start sampling may be approximated as occupying the center of the sampling domain. The Euclidean metric in the (3N‐6)‐dimensional conformational space decides which sample points form the boundaries of the sampling domain by their distance to the initial geometry in conformational space. The second goal is attained by consistent improvement of the kriging engine, and making sure that the test points are uniformly close to the training data, allowing for efficient interpolation. Note that we do not necessarily choose test points that are close to the training points. In other words, the training and test sets are constructed independently. By ensuring that the training data is uniformly distributed throughout the sampling domain, the average “distance” between an arbitrary test point and a training point will equal that between some other arbitrary test point and another training point. This guarantees no spurious predictions in under‐trained regions of conformational space. Of course, it is prudent to invoke some form of importance sampling, which yields a greater sampling density in more “important” regions of conformational space, but this issue has not been explored.

**Figure 3:**
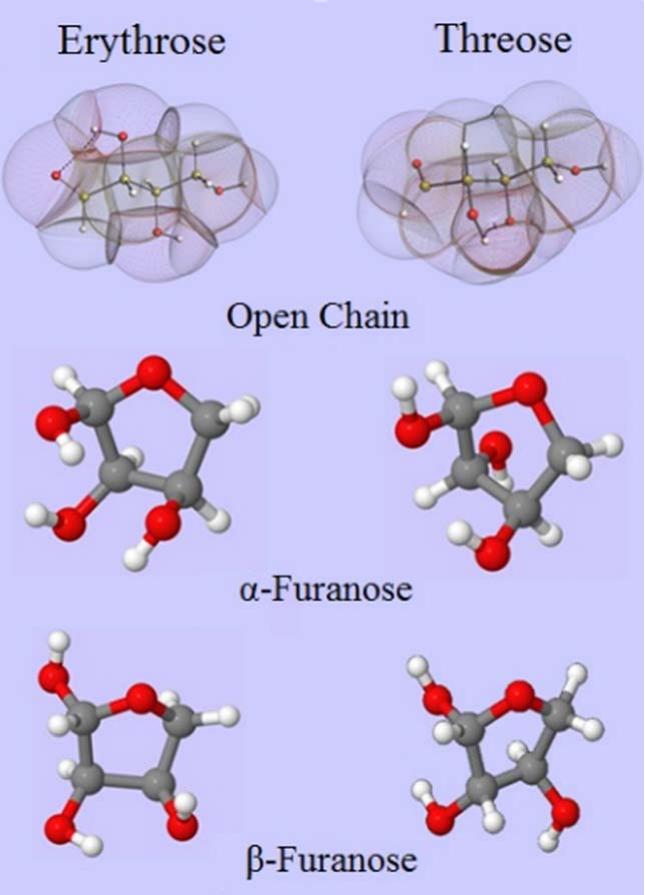
Comparison of erythrose and threose in the open chain (topological atoms and molecular graph) and in the ring configurations of α and β furanose (in traditional ball‐and‐stick representation). [Color figure can be viewed in the online issue, which is available at wileyonlinelibrary.com.]

We work on the tetrose diastereomers erythrose and threose, the smallest carbohydrates that adopt open chain and furanose forms. The particular conformations studied are given in Figure 3. Energetic minima were provided by Prof Alkorta, who had previously conducted a PES scan of these species,^44^ and are reported more thoroughly elsewhere.^45^ The chemical structures of these molecules are given in Figure 3. All geometries were subsequently optimized by the program GAUSSIAN03^46^ at the B3LYP/6‐311++G(d,p) level of theory. The Hessians were computed for each geometry, and utilized in the conformational sampling methodology we have described in the previous section, allowing for the output of 2000 geometries for each system. Single point calculations were performed on each sample at the B3LYP/apc‐1^47^ level of theory. The apc‐1 basis set (which is a polarization‐consistent (pc) double‐ζ plus polarization basis set with diffuse functions) was used for the DFT calculations, since this family of basis sets has been specifically optimized for DFT. The resultant wavefunctions are then passed on to program AIMAll,^48^ which calculates the atomic multipole moment according to QCT. We are only interested in the internal degrees of freedom of the molecular configurations.

Hence the atomic multipole moments must be expressed in an atomic local frame (ALF) rather than in the global frame. This procedure makes sure that the kriging focuses on the variation of atomic multipole moments within the molecule. Otherwise, when referring to the global frame (rather than the ALF), the three components of an atomic dipole moment, for example, vary upon rigid rotation of the whole molecule. Training for such a variation is useless. The same principle applies to atomic multipole moments of rank ℓ≥2. The details of the atomic local frame chosen for our work are outlined elsewhere.^49^ Briefly, the origin of the ALF is the nuclear position of the atom of interest, which is kriged. The heaviest (by atomic number, see Cahn‐Ingold‐Prelog rules) nucleus, which is directly bonded to the atom of interest, determines the ALF's x‐axis, the second heaviest determines the xy‐plane such that an orthogonal y‐axis can be installed. The three atoms involved in the ALF are described by the first three features: the distance between origin and x‐nucleus, the distance between origin and “y‐nucleus” and the corresponding suspended angle. The non‐ALF atoms are located by features that coincide with the familiar spherical coordinates (r, θ, φ), expressed with respect to the ALF. Kriging of each multipole moment (up to the hexadecapole moment) was performed for each atom by the in‐house program *FEREBUS 1.4* and models consisting of *N* training examples were generated. Atom‐atom interaction energies of 1‐5 and higher were computed in a test set of 200 arbitrary conformations, using the *ab initio* multipole moments, and compared to the interaction energies from multipole moments predicted by the kriging models. Errors are given in the form of so‐called S‐curves, which map the percentile of conformations within the test set, predicted up to a maximum error chosen, which is read off on the abscissa.

Here, we give a brief overview of how the force field we propose could be utilised within the context of MD. We limit the discussion to the evaluation of electrostatic interactions, but work is currently being undertaken within our group to establish the framework for an entire force field,^50^ which deviates considerably from the terms arising in a classical force field. The non‐electrostatic terms are also obtained via the QCT partitioning of molecular energy, originally derived from work in Ref. 51 and then elaborated in an approach called Interacting Quantum Atoms (IQA).^52^ At designated points over the course of a MD simulation, the conformational state of the system is evaluated. At this point, atomic multipole moments, up to the hexadecapole moment, can be extracted from the kriging models, and subsequently utilized for the evaluation of interatomic electrostatic interactions. In this way, we capture the conformational dependence of the atomic multipole moments. Separate kriging models are obtained for the non‐electrostatic terms, that is, the intra‐atomic energy (both kinetic^53^ and potential), the short‐range interatomic Coulomb energy not obtained by multipolar expansion, and the interatomic exchange energy.

The issue of coordinate frames needs further clarification because the Cartesian MD frame coordinate system is not the same as the local coordinate frame within which we have evaluated the atomic multipole moments (ALF). Prior to invoking a kriging model for the evaluation of multipole moments, the conformational state of the system must be converted from a Cartesian frame of reference to an ALF. From here, atomic multipole moments can be evaluated corresponding to the current state of the system. Previous work has derived the forces arising from the interactions of atomic multipole moments within the Cartesian frame of reference,^54^ which requires the partial derivatives of the multipole moments with respect to the ALF degrees of freedom. These terms have an analytical functional form, and so computation of the forces in the Cartesian frame of reference can be performed explicitly. These forces can subsequently be utilized by the standard MD procedure.

An embryonic workflow has been integrated within the MD package DL_POLY 4.0. Currently, an atomic kriging model is loaded into memory and the relevant data stored, followed by removal of the kriging model from memory. In this way, the dynamic memory requirements have not yet exceeded roughly 200 Mb, and are thus well within the capabilities of modern computational resources. Of course, memory management is crucial to the speed of the proposed methodology, and so will require a great deal of fine‐tuning. However, much speedup can undoubtedly be accomplished by a number of techniques, e.g. caching of regularly used quantities and parallel implementation.

Finally, it is too early to extensively comment on the computational cost of the current approach. It would be naïve to directly compare the flop count of the current force field with a traditional one without appreciating that (i) extra non‐nuclear point charges are needed to match the accuracy of multipole moments and the former propagate over long range, (ii) multipolar interactions drop off much faster than 1/r, depending on the rank of the interacting multipole moments, which depends on the interacting elements themselves (see extensive testing in the protein crambin^55^), (iii) the efficiency of the multipolar Ewald summation^56^ that is being implemented in DL_POLY 4.0, (iv) the dominance of monopolar interactions at long range (vast majority of interactions) and the (v) outstanding fine‐tuning of the kriging models at production mode. The current force field may well be an order of magnitude slower than a traditional force field. This estimate and the fact that the current force field contains electronic information invite one to compare its performance with on‐the‐fly *ab initio* calculations instead.

## Results and Discussion

### Single minimum

**Figure 4:**
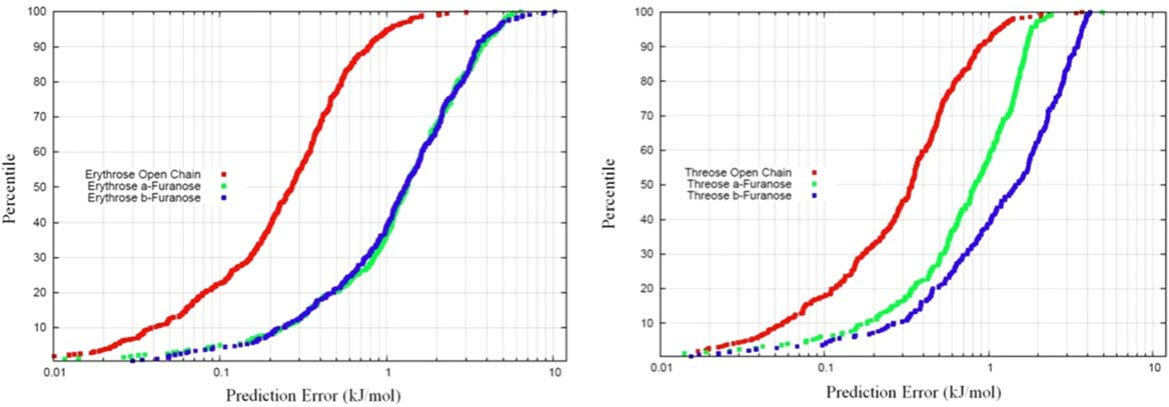
S‐curves corresponding to all erythrose (left) and threose (right) systems studied. The open chain forms are systematically better predicted than the corresponding furanose forms. [Color figure can be viewed in the online issue, which is available at wileyonlinelibrary.com.]

The lowest energy conformer for each system was chosen as an input structure for sampling. S‐Curves were subsequently generated for each of these training sets by the methodology outlined in the previous section. The S‐curves are given in Figure 4, and the accompanying mean errors in Table 1.

**Table 1:** Mean errors associated with the S‐curves given in Figure 4.

		Mean error (kJmol^−1^)	
	Open chain	α‐Furanose	β‐Furanose
Erythrose	0.27	1.32	1.30
Threose	0.34	0.83	1.47

The first point to notice is that the open chains for both erythrose and threose are modeled by kriging to a significantly better standard than the furanose forms. We may, however, immediately attribute this to the number of 1‐5 and higher interactions occurring in these systems. For both open chains, 25 interactions are required to be evaluated for comparison to the energies produced from the *ab initio* multipole moments. The numbers of interactions requiring evaluation for the furanose forms comes to 39, which is virtually twice the amount evaluated in the open chain forms. We would subsequently expect a proportional relationship between the number of interactions required for evaluation and the mean error attributed to the kriging model. Whilst we see this to be roughly true when comparing the errors on the threose open and α‐furanose forms, the errors appear disproportionately higher for the other systems.

Kriging is an interpolative technique, and so is not suited for extrapolation. However, we point out that the kriging engine is still predictive for extrapolation‐ in this case, the prediction falls to the mean value of the function. Obviously this is not ideal for highly undulatory functions. However, considering how the atomic multipole moments do not fluctuate over vast ranges, the mean will often represent a respectable prediction to the function value. The kriging model can be refined in an iterative fashion, whereby extrapolation points are added to the training set. This is a commonly used technique in the field of machine learning. Whilst not currently implemented within our methodology, the iterative protocol is a technique which is currently being explored.

Regardless of the problem encountered in the above, we see that it is easily remedied by strategic sampling of conformational space. In fact, these problems are ubiquitous to machine learning techniques, and have been encountered in studies which attempt to implement neural networks to predict a PES.^57^ In this field, the problems have been solved to some extent by making the prediction engine issue a warning to the user that a point is being predicted which lies outside of the training set. This proves to be advantageous as the user may then recognize that the point should be included within the training set as it obviously lies within an accessible portion of conformational space. The point can then be included within the training set, and one can generate refined models by undergoing this process iteratively.

### Training set dependency

**Figure 5:**
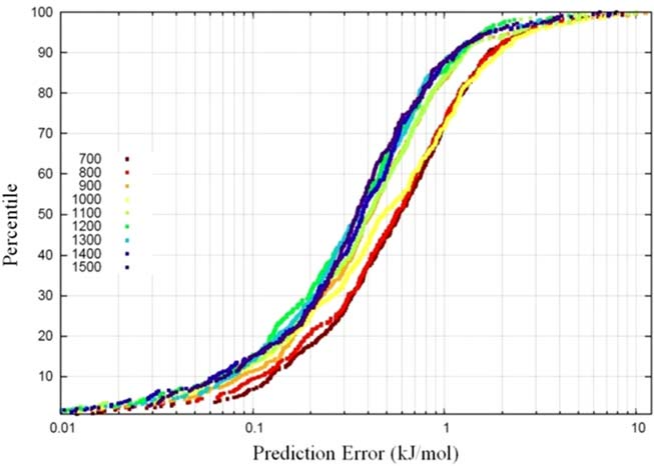
S‐curves for erythrose open chain at various training set sizes. Note the progression of the S‐curves towards the lower prediction errors as the training set size increases. However, owing to the logarithmic abscissa, this does not correspond to a uniform enhancement of a kriging model given a consistently larger training set size. [Color figure can be viewed in the online issue, which is available at wileyonlinelibrary.com.]

We start by discussing the effects of increasing the training set size on the prediction error for a kriging model. For this purpose, we use the erythrose open chain system owing to its higher conformational flexibility, which we assume amplifies the effects of training set size. Kriging models were generated for this system with training sets ranging from 700 to 1500 sampling points, in increments of 100. The same test set (of 200 points) was reserved for prediction by all models. The S‐curves for this are given in Figure 5.

As expected, the prediction errors of the S‐curves in Figure 5 systematically decrease as the training set size is increased. In other words, the S‐curves move to the left with increasing training set size, although this is not true for all parts of the S‐curves because they clearly intersect in many places. Overall the uniform increments of 100 in training set size are not matched by equal uniform strides of improvement in S‐curve shape and position. An alternative way to gauge the improvement in prediction with increasing training set size is monitoring the average prediction error for each S‐curve. This value cannot be read off for an S‐curve in Figure 5 but can be easily calculated.

**Figure 6:**
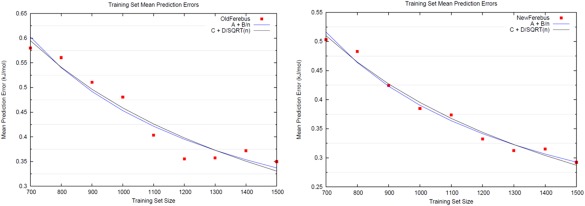
Mean prediction errors associated with S‐curves for erythrose open chain as the training set size is increased. With the old kriging engine (left), a distinct plateau formed after roughly 1200 training examples, corresponding to no further improvement in the kriging model despite additional training points. However, the new kriging engine (right) appears to avoid premature plateauing, with additional kriging model improvement at higher training set sizes. Regression fits of the FEREBUS errors against training set size, of functional forms A + B/*n* (blue) and C + D/ $\sqrt{n}$ (black), where *n* is the training set size, are also given. [Color figure can be viewed in the online issue, which is available at wileyonlinelibrary.com.]

Figure 6 plots the average prediction error for each S‐curve against increasing training set size: red for “Old FEREBUS” and blue for “New FEREBUS,” a development version of our kriging engine, which differs in a number of ways to the “Old FEREBUS.” We include both “Old FEREBUS” and “New FEREBUS” data to establish whether any functional forms of average prediction error against increasing training set size are conserved with respect to improvements in the engine. The “Old FEREBUS” data show a plateau in the average error (left pane) at a training set size of about 1200, after an initial decrease in this error. This plateau would be rather problematic, as it implies some maximum efficiency of the kriging engine, beyond which there is no reward for an extension of the training set. However, this is not the case for the New FEREBUS data (right pane).

Learning theory states that for a machine learning method of this type (kriging), the mean prediction error should decrease asymptotically toward zero, with functional form *A + B/n* or *C + D/* $\sqrt{n}$, where *n* is the training set size, and *A, B, C,* and *D* are fitted constants. Figure 6 plots these asymptotes, where (A_old_ = 0.105; B_old_ = 348.11) and (C_old_ = −0.293; D_old_ = 22.06), as determined by regression analysis against the Old FEREBUS data, each with *R* ^2^ coefficients of 0.93. Similarly, for the New FEREBUS data, constants of (A_new_ = 0.097; B_new_ = 293.38) and (C_new_ = −0.193; D_new_ = 18.60) were obtained. These fitted asymptotes both possess *R* ^2^ values of 0.98. As such, we conclude that the decay of the mean prediction error of our machine learning method possesses, as yet, inconclusive functional form.

The results in Figure 6 are consistent with the behavior seen in similar interpolation methods^58^: for an infinite training set size, the mean prediction error will asymptote to zero. However, for the methodology to remain computationally feasible, some finite training set size will of course be required.

So, for example, we find that for a mean prediction error of 0.3 kJ mol^−1^, the training set would require about 1450 sample points for either functional form taken as the decay of the prediction error, i.e. *A + B/n* or *C + D/* $\sqrt{n}$.

A comment on the nature of the average error is in place here. In principle, the prediction error consists of the sum of the estimation error and the approximation error. From learning theory, one expects the estimation error only to go to zero. The bias–variance decomposition of a learning algorithm's error also contains a quantity called the irreducible error, resulting from noise in the problem itself. This error has been investigated some time ago in the context of tests on kriging of ethanol multipole moments^[41]^ and is caused by the small noise generated by the integration quadrature of the atomic multipole moments. Second, any bias caused by an inherent error in the *ab initio* method used, compared to the best method available (e.g. CCSD(T) with a complete basis set), is not relevant in our error considerations. The reason is that we always assess the performance of kriging training against the (inevitably approximate) *ab initio* at hand, which we refer to the source of “the” *ab initio* data.

### Multiple minima

The amount of conformational space available to molecular systems reaches levels which are entirely unfeasible for systematic sampling as the number of atoms increases. As such, it becomes all the more prudent to obtain an efficient sampling scheme for our purposes. As we have mentioned, our sampling methodology is limited to local conformational exploration about some given input geometry, since the PES about that point is approximated as a harmonic well. As such, to thoroughly explore conformational space, our methodology requires the usage of a number of such starting geometries. Then, the molecular PES is approximated by a number of harmonic wells. If the input geometries are sufficiently close to one another, the wells will overlap, and the PES may be explored seamlessly. Non‐equilibrium normal mode conformational sampling has also been demonstrated in a recent publication.^59^ This advance will facilitate a more thorough sampling of these higher energy parts potential energy surfaces. The validation of this methodology is presented in Part B of Supporting Information.

**Figure 7:**
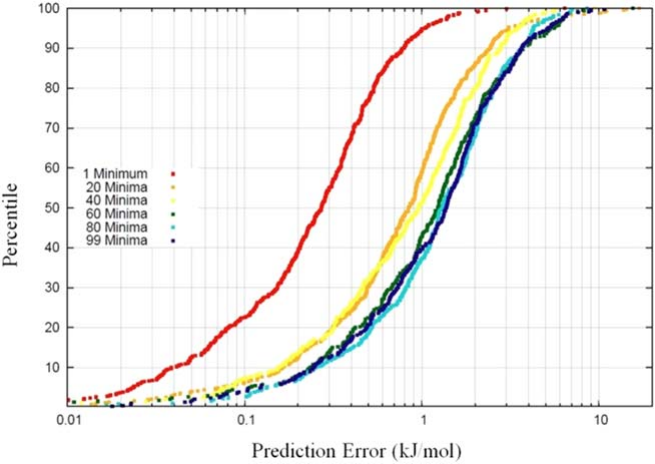
S‐curves depicting the power of a kriging model as more energetic minima are utilized for conformational sampling. As the number of minima utilized increases, the S‐curves tend toward higher prediction errors. The kriging models which underlie these S‐curves have a fixed training set size of 700. [Color figure can be viewed in the online issue, which is available at wileyonlinelibrary.com.]

For the open chain form of erythrose, 174 energetic minima were found by an exhaustive search of conformational space. Figure 7 plots the S‐curves obtained for samples which have been generated from different numbers of up to 99 minima. The S‐curves display increasingly poor prediction results as the number of starting minima increases. The actual mean errors for these S‐curves are summarized in Table 2. This trend has a logical interpretation. As the number of seeding structures increases, the sampled conformational space grows in size. Given a fixed kriging model size, the sampling density therefore decreases. The kriging model then deviates from the true analytical function, and the results from predictions deteriorate.

**Table 2:** Mean errors corresponding to the S‐curves depicted in Figure 7.

Number of minima	1	20	40	60	80	99
Mean prediction error (kJmol^−1^)	0.27	0.85	0.9	1.23	1.30	1.37

Note the “bunching” of prediction error when 60 energetic minima or higher are used as seeds for the conformational sampling.

Of course, thorough sampling of conformational space is an issue for parameterizing any force field, and by no means one that is resultant from our methodology. We may overcome this issue in two ways. The first is the ongoing improvement of our kriging engine to deal with larger training sets comprising more molecular configurations. The second is by undertaking sampling with only a subset of the energetic minima that are available. This is all the more valid an approach if most of the minima are very high in energy relative to the lowest‐lying minima. These regions of conformational space will be accessed very infrequently during the course of a MD simulation, and so may be sampled much more coarsely. This selective sampling is quite readily employed, and has been discussed at length in the literature. For example, Brooks and Karplus^60^ found that a comprehensive sampling of conformational space for bovine pancreatic trypsin inhibitor could be achieved by evolving only the lowest frequency normal modes of motion. Needless to say, this is readily accomplished by our sampling methodology.

## Conclusion

We have demonstrated that the atomic multipole moments of a set of carbohydrates are amenable to the machine learning technique kriging. Whilst this has been done in the past for a variety of chemical species including naturally occurring amino acids, this is the first foray into the field of glycobiology. Kriging is able to capture the conformational dependence of the multipole moments and make predictions, such that the error in the electrostatic energy relative to that derived from *ab initio* data is encouraging, given the popular aim is to obtain errors below 4 kJ mol^−1^. Indeed, the presented methodology is immediately extensible to any term arising in an energetic decomposition of a system. If some quantity is conformationally dependent, then the dependence can be modelled by kriging. As such, an entire force field can be parameterized by the current methodology, reproducing *ab initio* quantities for use in classical MD. This route is preferable to the computationally intensive approach of *ab initio* MD.

## Supporting information

Supporting Information

## References

[1] M. L. DeMarco , R. J. Woods , Glycobiology 2008, 18, 426. 18390826 10.1093/glycob/cwn026 PMC4203483

[2] C. H. Faerman , S. L. Price , J. Am. Chem. Soc. 1990, 112, 4915.

[3] E. Juaristi , G. Cuevas , The Anomeric Effect; CRC press, 1994.

[4] B. Lachele Foley , M. B. Tessier , R. J. Woods , WIREs Comput. Mol. Sci. 2012, 2, 652. 10.1002/wcms.89 PMC4270206 25530813

[5] V. R. Rao , Conformation of Carbohydrates; CRC Press, 1998.

[6] K. N. Kirschner , R. J. Woods , Proc. Natl. Acad. Sci. USA 2001, 98, 10541. 11526221 10.1073/pnas.191362798 PMC58501

[7] E. Fadda , R. J. Woods , Drug Discov. Today 2010, 15, 596. 20594934 10.1016/j.drudis.2010.06.001 PMC3936463

[8] J. Kaminský , J. Kapitán , V. Baumruk , L. Bednárová , P. Bour , J. Phys. Chem. A 2009, 113, 3594. 19309136 10.1021/jp809210n

[9] J. Cheeseman , M. S. Shaik , P. L. A. Popelier , E. W. Blanch , J. Am. Chem. Soc. 2011, 133, 4991. 21401137 10.1021/ja110825z

[10] K. N. Kirschner , A. B. Yongya , S. M. Tschampel , J. Gonzalez‐Outeirino , C. R. Daniels , B. Lachele Foley , R. J. Woods , J. Comp. Chem. 2008, 29, 622. 17849372 10.1002/jcc.20820 PMC4423547

[11] I. T. Todorov , W. Smith , K. Trachenko , M. T. Dove , J. Mater. Chem. 2006, 16, 1911.

[12] A. M. Salisburg , A. L. Deline , K. W. Lexa , G. C. Shields , K. N. Kirschner , J. Comput. Chem. 2009, 30, 910. 18785152 10.1002/jcc.21099

[13] M. L. DeMarco , R. J. Woods , Glycobiology 2009, 19, 344. 19056784 10.1093/glycob/cwn137 PMC2733776

[14] M. L. DeMarco , R. J. Woods , Mol. Immunol. 2011, 49, 124. 21924775 10.1016/j.molimm.2011.08.003 PMC3252744

[15] M. B. Tessier , M. L. DeMarco , A. B. Yongye , R. J. Woods , Mol. Simul. 2008, 34, 349. 22247593 10.1080/08927020701710890 PMC3256582

[16] M. Basma , S. Sundara , D. Çalgan , T. Vernali , R. J. Woods , J. Comput. Chem. 2001, 22, 1125. 17882310 10.1002/jcc.1072 PMC1986576

[17] S. Cardamone , T. J. Hughes , P. L. A. Popelier , Phys. Chem. Chem. Phys. 2014, 16, 10367. 24741671 10.1039/c3cp54829e

[18] R. F. W. Bader , Atoms in Molecules. A Quantum Theory; Oxford University Press: Oxford, Great Britain, 1990.

[19] P. L. A. Popelier , Quantum Chemical Topology: On Descriptors, Potentials and Fragments. In Drug Design Strategies: Computational Techniques and Applications, L. Banting, T. Clark, Eds.; Roy. Soc. Chem., Great Britain: Cambridge, Vol. 20; 2012; Chapter 6, pp. 120–163.

[20] P. L. A. Popelier , On Quantum Chemical Topology. In Challenges and Advances in Computational Chemistry and Physics dedicated to “Applications of Topological Methods in Molecular Chemistry”; Alikhani E., Chauvin R., Lepetit C., Silvi B., Eds.; Springer: Germany, 2015.

[21] P. L. A. Popelier , The Quantum Theory of Atoms in Molecules, In The Nature of the Chemical Bond Revisited; Frenking G., Shaik S., Eds.; Wiley‐VCH, Chapter 8, 2014; pp. 271–308.

[22] P. L. A. Popelier , É. A. G. Brémond , Int. J. Quant. Chem. 2009, 109, 2542.

[23] D. T. I. Nakazato , E. L. de Sa , R. L. A. Haiduke , Int. J. Quant. Chem. 2010, 110, 1729.

[24] B. Courcot , A. J. Bridgeman , Int. J. Quant. Chem. 2010, 110, 2155.

[25] S. Saha , R. K. Roy , P. W. Ayers , Int. J. Quant. Chem. 2009, 109, 1790.

[26] T. Verstraelen , P. W. Ayers , V. Van Speybroeck , M. Waroquier , J. Chem. Theory Comput. 2013, 9, 2221. 26583716 10.1021/ct4000923

[27] C. F. Matta , R. J. Boyd , The Quantum Theory of Atoms in Molecules; Wiley, 2007.

[28] N. O. J. Malcolm , P. L. A. Popelier , J. Comp. Chem. 2003, 24, 437. 12594786 10.1002/jcc.10203

[29] P. L. A. Popelier , Mol. Phys. 1996, 87, 1169.

[30] P. L. A. Popelier , L. Joubert , D. S. Kosov , J. Phys. Chem. A 2001, 105, 8254.

[31] E. F. F. Rodrigues , E. L. de Sa , R. L. A. Haiduke , Int. J. Quant. Chem. 2008, 108, 2417.

[32] S. M. Kandathil , T. L. Fletcher , Y. Yuan , J. Knowles , P. L. A. Popelier , J. Comput. Chem. 2013, 34, 1850. 23720381 10.1002/jcc.23333

[33] D. G. Krige , J. Chem . Metall. Mining Soc. South Africa 1951, 52, 119.

[34] D. R. Jones , J. Global Optim. 2001, 21, 345.

[35] C. E. Rasmussen , C. K. I. Williams , Gaussian Processes for Machine Learning; The MIT Press: Cambridge, USA, 2006.

[36] C. M. Handley , G. I. Hawe , D. B. Kell , P. L. A. Popelier , Phys. Chem. Chem. Phys. 2009, 11, 6365. 19809668 10.1039/b905748j

[37] J. Kennedy , R. C. Eberhart , Proc. IEEE Int. Conf. on Neural Networks 1995, 4, 1942.

[38] T. J. Hughes , S. M. Kandathil , P. L. A. Popelier , Spectrochimica Acta A 2015, 136, 32. 10.1016/j.saa.2013.10.059 24274986

[39] T. Fletcher , S. J. Davie , P. L. A. Popelier , J. Chem. Theory Comput. 2014, 10, 3708. 26588516 10.1021/ct500416k

[40] M. J. L. Mills , P. L. A. Popelier , Theor. Chem. Acc. 2012, 131, 1137.

[41] M. J. L. Mills , P. L. A. Popelier , Comput. Theor. Chem. 2011, 975, 42.

[42] J. W. Ochterski , Vibrational Analysis in Gaussian. *Vibrational Analysis in Gaussian, Available at*: http://www.gaussian.com/g_whitepap/vib.htm, 1999.

[43] W. H. Press , B. P. Flannery , S. A. Teucholsky , W. T. Vetterling , Numerical Recipes, 2nd ed; Cambridge University Press: Cambridge, 1992.

[44] I. Alkorta , P. L. A. Popelier , Carbohydr. Res. 2011, 346, 2933. 22063503 10.1016/j.carres.2011.10.013

[45] L. M. Azofra , I. Alkorta , J. Elguero , P. L. A. Popelier , Carbohydr. Res. 2012, 358, 96. 22841585 10.1016/j.carres.2012.06.011

[46] GAUSSIAN03 M. J. Frisch , G. W. Trucks , H. B. Schlegel , G. E. Scuseria , M. A. Robb , J. R. Cheeseman , J. A. J. Montgomery , J. T. Vreven , K. N. Kudin , J. C. Burant , J. M. Millam , S. S. Iyengar , J. Tomasi , V. Barone , B. Mennucci , M. Cossi , G. Scalmani , N. Rega , G. A. Petersson , H. Nakatsuji , M. Hada , M. Ehara , K. Toyota , R. Fukuda , J. Hasegawa , M. Ishida , T. Nakajima , Y. Honda , O. Kitao , H. Nakai , M. Klene , X. Li , J. E. Knox , H. P. Hratchian , J. B. Cross , C. Adamo , J. Jaramillo , R. Gomperts , R. E. Stratmann , O. Yazyev , A. J. Austin , R. Cammi , C. Pomelli , J. W. Ochterski , P. Y. Ayala , K. Morokuma , G. A. Voth , P. Salvador , J. J. Dannenberg , V. G. Zakrzewski , S. Dapprich , A. D. Daniels , M. C. Strain , O. Farkas , D. K. Malick , A. D. Rabuck , K. Raghavachari , J. B. Foresman , J. V. Ortiz , Q. Cui , A. G. Baboul , S. Clifford , J. Cioslowski , B. B. Stefanov , G. Liu , A. Liashenko , P. Piskorz , I. Komaromi , R. L. Martin , D. J. Fox , T. Keith , M. A. Al‐Laham , C. Y. Peng , A. Nanayakkara , M. Challacombe , P. M. W. Gill , B. Johnson , W. Chen , M. W. Wong , C. Gonzalez , J. A. Pople , In Gaussian, Inc., Pittsburgh PA, 2003, USA.

[47] F. Jensen , J. Chem. Phys. 2002, 117, 9234.

[48] T. A. Keith , AIMAll. 11.04.03 ed.; aim.tkgristmill.com, 2011.

[49] Y. Yuan , M. J. L. Mills , P. L. A. Popelier , J. Mol. Model. 2014, 20, 2172. 24633774 10.1007/s00894-014-2172-1

[50] P. L. A. Popelier , Int. J. Quant. Chem. 2015, 115, 1005.

[51] P. L. A. Popelier , D. S. Kosov , J. Chem. Phys. 2001, 114, 6539.

[52] M. A. Blanco , A. M. Pendas , E. Francisco , J. Chem. Theor. Comput. 2005, 1, 1096. 10.1021/ct0501093 26631653

[53] T. L. Fletcher , S. M. Kandathil , P. L. A. Popelier , Theor. Chem. Acc. 2014, 133, 1499:1.

[54] M. J. L. Mills , P. L. A. Popelier , J. Chem. Theory Comput. 2014, 10, 3840. 26588529 10.1021/ct500565g

[55] Y. Yuan , M. J. L. Mills , P. L. A. Popelier , J. Comp. Chem. 2014, 35, 343. 24449043 10.1002/jcc.23469

[56] H. A. Boateng , I. T. Todorov , J. Chem. Phys. 2015, 142, 034117. 25612699 10.1063/1.4905952

[57] J. Behler , Phys. Chem. Chem. Phys. 2011, 13, 17930. 21915403 10.1039/c1cp21668f

[58] M. A. Collins , Theor. Chem. Acc. 2002, 108, 313.

[59] T. J. Hughes , S. Cardamone , P. L. A. Popelier , J. Comp. Chem. 2015, 36, 1844. 26235784 10.1002/jcc.24006 PMC4973712

[60] B. Brooks , M. Karplus , Proc. Natl. Acad. Sci. 1983, 80, 6571. 6579545 10.1073/pnas.80.21.6571 PMC391211

