# Supplementary material for: Prediction of conformationally dependent atomic multipole moments in carbohydrates
Source: J Comput Chem. 2015 Nov 8;36(32):2361–73. doi: 10.1002/jcc.24215 (PMC5031233; doi:10.1002/jcc.24215)
Supplement: Supplementary file 1 — Supporting Information [file JCC-36-2361-s001.docx]

**Supplementary Information**

## Salvatore Cardamone and Paul L. A. Popelier*

**Part A**

**Stationary Point Normal Modes**

**A1. Overall Derivation**

**A1.1 Kinetic Energy**

We begin with a Cartesian molecular configuration, $\boldsymbol{x}=\left[ x_{1},\ldots,x_{3N} \right]^{\top}$, and define a difference coordinate, $\Delta\boldsymbol{x}$, relative to some arbitrary configuration, $\boldsymbol{x}^{*}=\left[ x_{1}^{*},\ldots,x_{3N}^{*} \right]^{\top}$, such that

| $\Delta\boldsymbol{x}=\boldsymbol{x-}\boldsymbol{x}^{\boldsymbol{*}}\boldsymbol{=}\left[ x_{1}-x_{1}^{*},\ldots,x_{3N}-x_{3N}^{*} \right]^{\top}=\left[ \Delta x_{1},\ldots,\Delta x_{3N} \right]^{\top}$ | (S1) |
| --- | --- |

We may do this without loss of generality for the following argument because we constrain $\boldsymbol{x}^{*}$ to be static. The classical expression for the kinetic energy of a system is then given by

| $T\left( \Delta\dot{x}_{1},\ldots,\Delta\dot{x}_{3N} \right)= \sum_{i}^{3N} \frac{m_{i}}{2}\left( \frac{d}{dt}\Delta x_{i} \right)^{2}$ | (S2) |
| --- | --- |

where $m_{i}$ is the mass of the atom to which the $i^{th}$ degree of freedom belongs. So far everything has been expressed in Cartesian coordinates but it is convenient to introduce mass-weighted (Cartesian) coordinates $q_{i}$,

| $q_{i}=\Delta x_{i}\sqrt{m_{i}}$ | (S3) |
| --- | --- |

and substitution of its time derivative into *Equation* *S2*, we obtain

| $T\left( \dot{q}_{1},\ldots,\dot{q}_{3N} \right)=\frac{1}{2}\sum_{i}^{3N} \left( \frac{d}{dt}q_{i} \right)^{2}=\frac{1}{2}\sum_{i}^{3N} \dot{q}_{i}^{2}$ | (S4) |
| --- | --- |

where the conventional dot notation has been adopted to represent the time derivative.

**A1.2 Potential Energy**

The potential energy corresponding to a state, $V\left( \boldsymbol{x} \right)=V\left( x_{1},\ldots,x_{3N} \right)$ is given by a Taylor series about the predefined configuration $\boldsymbol{x}^{\boldsymbol{*}}$, leading to

| $2V\left( x_{1},\ldots,x_{3N} \right)=2V_{0}\left( x_{1}^{*},\ldots,x_{3N}^{*} \right)+ 2\sum_{i}^{3N} \left( x_{i}-x_{i}^{*} \right) \left. \frac{\partial V}{\partial x_{i}} \right\vert_{\boldsymbol{x}^{\boldsymbol{*}}}+ \sum_{i,j}^{3N} \left( x_{i}-x_{i}^{*} \right)\left( x_{j}-x_{j}^{*} \right)\left. \frac{\partial^{2}V}{\partial x_{i}\partial x_{j}} \right\vert_{\boldsymbol{x}^{\boldsymbol{*}}}+\ldots$ | (S5) |
| --- | --- |

The derivative factors are actually constants because they are evaluated at $\boldsymbol{x}^{\boldsymbol{*}}$ (a point to be kept in mind when differentiating further). *Equation S6* introduces two definitions

| $\left. \frac{\partial V}{\partial x_{i}} \right\vert_{\boldsymbol{x}^{*}}=J_{i}^{'} \mathrm{and} \left. \frac{\partial^{2}V}{\partial x_{i}\partial x_{j}} \right\vert_{\boldsymbol{x}^{*}}=H_{ij}^{'}$ | (S6) |
| --- | --- |
| such that |  |
| $2V\left( x_{1},\ldots,x_{3N} \right)=2V_{0}\left( x_{1}^{*},\ldots,x_{3N}^{*} \right)+ 2\sum_{i}^{3N} \left( x_{i}-x_{i}^{*} \right) J_{i}^{'}+ \sum_{i,j}^{3N} \left( x_{i}-x_{i}^{*} \right)\left( x_{j}-x_{j}^{*} \right)H_{ij}^{'}+\ldots$ | (S7) |

The first-order and second-order spatial derivatives of the potential energy *V* correspond to elements of the Jacobian^[[1]](#footnote-1)^ and Hessian, respectively. By choosing $\boldsymbol{x}^{*}$ such that it occupies a stationary point on the potential energy surface, we are free to set $V\left( \boldsymbol{x}^{\boldsymbol{*}} \right)=0$. Additionally, the first derivative (Jacobian) term in the Taylor series necessarily goes to zero at this stationary point. By omitting all terms strictly higher than the second order, we obtain

| $2V\left( x_{1},\ldots,x_{3N} \right)= \sum_{i,j}^{3N} \left( x_{i}-x_{i}^{*} \right)\left( x_{j}-x_{j}^{*} \right)H_{ij}^{'}$ | (S8) |
| --- | --- |

It is useful to express the potential energy in the same coordinates as those used for the kinetic energy. This can be achieved using *Equation S3* and *Equation S9*, which follows from *Equation S3*,

| $\frac{\partial}{\partial q_{i}}=\frac{1}{\sqrt{m_{i}}}\frac{\partial}{\partial\left( x_{i}-x_{i}^{*} \right)}=\frac{1}{\sqrt{m_{i}}}\frac{\partial}{\partial x_{i}}$ | (S9) |
| --- | --- |

such that, when both substituted in *Equation S8* (and using *Equation S6*), we obtain

| $2V\left( x_{1},\ldots,x_{3N} \right)= \sum_{i,j}^{3N} \Delta x_{i}\Delta x_{j}\left. \frac{\partial^{2}V}{\partial x_{i}\partial x_{j}} \right\vert_{\boldsymbol{x}^{*}}=\sum_{i,j}^{3N} {\frac{q_{i}q_{j}}{\sqrt{m_{i}m_{j}}}\sqrt{m_{i}m_{j}}}\left. \frac{\partial^{2}V}{\partial q_{i}\partial q_{j}} \right\vert_{\boldsymbol{0}}= \sum_{i,j}^{3N} H_{ij}q_{i}q_{j}=2V\left( q_{1},\ldots,q_{3N} \right)$ | (S10) |
| --- | --- |

where we hereafter call the mass-weighted elements of the Hessian, denoted $H_{ij}=\frac{1}{\sqrt{m_{i}m_{j}}}H_{ij}^{'}$ and $H_{ij}=\left. \frac{\partial^{2}V}{\partial q_{i}\partial q_{j}} \right|_{\boldsymbol{0}}\boldsymbol{=}\frac{1}{\sqrt{m_{i}m_{j}}}\left. \frac{\partial^{2}V}{\partial x_{i}\partial x_{j}} \right|_{\boldsymbol{x}^{\boldsymbol{*}}}$

**A1.3 Equations of Motion**

Substituting *Equations S4* and *S10* into *Equation S11*, which are the Euler-Lagrange equations of motion,

| $\frac{d}{dt}\frac{\partial T}{\partial\dot{q}_{k}}+\frac{\partial V}{\partial q_{k}}=0 \forall k=1,2,\ldots,3N$  leads to | (S11) |
| --- | --- |
| $\frac{d}{dt}\frac{\partial}{\partial\dot{q}_{k}} \left( \frac{1}{2}\sum_{i}^{3N} \dot{q}_{i}^{2} \right) + \frac{\partial}{\partial q_{k}}\left( \frac{1}{2}\sum_{i,j}^{3N} H_{ij}q_{i}q_{j} \right)=\frac{d}{dt}\left( \frac{1}{2}\sum_{i}^{3N} \frac{\partial}{\partial\dot{q}_{k}} \dot{q}_{i}^{2} \right) + \left( \frac{1}{2}\sum_{i,j}^{3N} {\frac{\partial}{\partial q_{k}}(H}_{ij}q_{i}q_{j}) \right)$ $=\frac{d}{dt}\left( \sum_{i}^{3N} \delta_{ik}\dot{q}_{i} \right)+\frac{1}{2}\sum_{i,j}^{3N} H_{ij}q_{i}\frac{\partial q_{j}}{\partial q_{k}}+\frac{1}{2}\sum_{i,j}^{3N} H_{ij}q_{j}\frac{\partial q_{i}}{\partial q_{k}}$ $=\frac{d\dot{q}_{k}}{dt}+\frac{1}{2}\sum_{i,j}^{3N} H_{ij}q_{i}\delta_{jk}+\frac{1}{2}\sum_{i,j}^{3N} H_{ij}q_{j}\delta_{ik}=\frac{d^{2}}{dt^{2}}q_{k}+\frac{1}{2}\sum_{i}^{3N} H_{ik}q_{i}+\frac{1}{2}\sum_{j}^{3N} H_{kj}q_{j}$ $=\frac{d^{2}}{dt^{2}}q_{k}+ \sum_{i}^{3N} H_{ik}q_{i}=0$ | (S12) |

where $\delta_{ij}$ is the Kronecker delta, equal to 1 if $i=j$ and 0 otherwise. We have invoked the symmetric nature of the Hessian $H_{ij}=H_{ji}$ and the fact that the last two sums are identical because $i$ and $j$ are dummy indices and therefore $j$ can be written as $i$.

We have thus obtained a second-order homogeneous differential equation (HDE), the solution of which is a simple superposition of sinusoids of angular frequency $\omega$ and amplitudes $A_{k}$ and $B_{k}$ for the $k^{th}$ equation of motion,

| $q_{k}\left( t \right)=A_{k}\cos\left( \omega t \right)+ B_{k}\sin\left( \omega t \right)$ | (S13) |
| --- | --- |

We choose to use the more compact notation of a single sinusoid with a phase factor, $\phi$

| $q_{k}\left( t \right)=A_{k}\cos\left( \omega t+\phi\right)$ | (S14) |
| --- | --- |

Placing *Equation S14* into *Equation S12*, we obtain

| $\frac{d^{2}}{dt^{2}}A_{k}\cos\left( \omega t+\phi\right)+\sum_{i}^{3N} H_{ik}A_{i}\cos\left( \omega t+\phi\right)=0$ | (S15) |
| --- | --- |
| $-\omega^{2}A_{k}\cos\left( \omega t+\phi\right)+ \sum_{i}^{3N} H_{ik}A_{i}\cos\left( \omega t+\phi\right)=0$ | (S16) |

The next step involves the cancellation of the factor $\cos\left( \omega t+\phi\right)$ in each term. However, this action places a constraint on the solution of *Equation S14*, in case this factor is equal to zero, or when $\omega t+\phi=(2n+1)\pi/2$ where $n \in\mathbb{N}$. However, in that case we recover that $q_{k}\left( t \right)=0$ at the stationary point, which satisfies *Equation S16*. Continuing with the case of non-zero $\cos\left( \omega t+\phi\right)$

| $-\omega^{2}A_{k}+ \sum_{i}^{3N} H_{ik}A_{i}=0 \therefore\sum_{i}^{3N} A_{i}\left( H_{ik}- \omega^{2}\delta_{ik} \right)=0$ | (S17) |
| --- | --- |

This equation constitutes an eigensystem for which there exist $3N$ values of $\omega$, which give rise to non-trivial solutions for the $q_{k}\left( t \right)$, i.e. where $A_{k}\neq0$. These solutions may be found by diagonalisation of the mass-weighted Hessian, the eigenvalues of which correspond to the $3N$ frequencies, as may be seen by evaluation of the factor in parentheses in *Equation S17*. Of course, this procedure is typically carried out in an internal coordinate basis, which renders six of the $3N$ degrees of freedom invariant. This then results in six of the eigenvalues of the mass-weighted Hessian being equal to zero, corresponding to the frequencies of the three global translational and three global rotational degrees of freedom. Note that, from here on, the index $k$ runs from $1$ to $3N-6$, because we disregard those normal modes with a frequency of zero.

**Part B**

**Rationale and Validation of Normal Modes Conformational Sampling**

A sampling scheme that is reliant upon normal modes inhibits the sampling of those degrees of freedom typically deemed as “flexible”, for example, the torsional motion of a dihedral angle. A number of stable energetic minima corresponding to these torsional degrees of freedom can possibly exist. The free rotation of a methyl group in ethane, for example, possesses three such energetic minima, corresponding to the so-called “staggered” configurations. However, the harmonic potential-well approximation for the potential energy of dihedral angle cannot capture the three stable configurations. This situation is summarised in Figure S1.


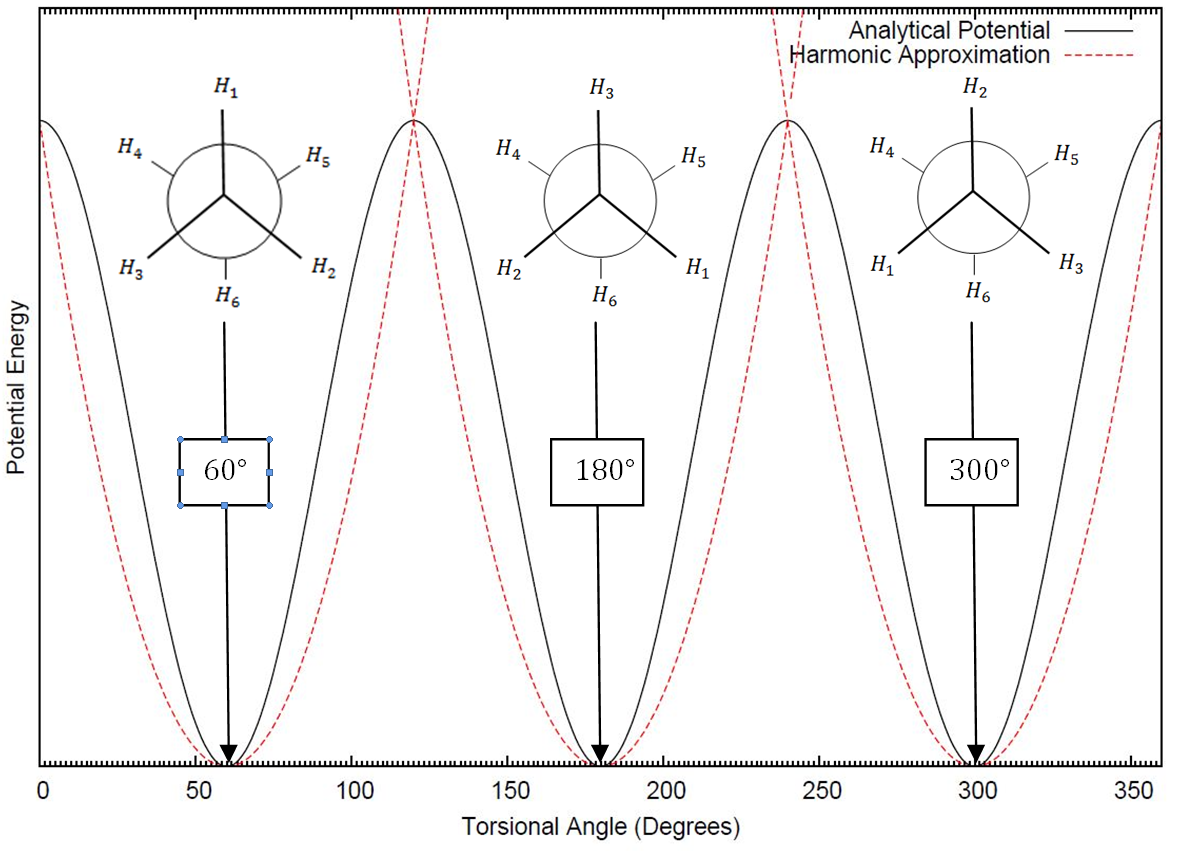


**Figure S1.** Analytical potential for the free rotation of the methyl group in ethane (black line). Three energetic minima corresponding to the depicted Newman projections are clearly marked. The analytical potential is well approximated by a series of three overlapping local harmonic wells (red dashed lines). This demonstrates that sufficiently coarse torsional sampling can be accomplished by the normal mode conformational sampling methodology we have proposed, given that the three local minima are utilised as seeding geometries.

To overcome the obstacle of a single potential well not covering the whole potential, one can select a number of energetic minima as seeds from which to sample. In the discussion of the ethane example, we ignore its internal symmetry, for sake of argument. Here, the seed selection involves the three “staggered” conformations as seeding structures, and subsequently approximating the full PES as a series of three overlapping harmonic wells. In this way, the limited amount of torsional motion allowed by a single harmonic approximation is overcome. The harmonic potential wells actually permit for a greater level of local flexibility relative to the analytical potential. Indeed, the harmonic potential wells possess a shallower curvature up to the maxima of the analytical potential, from which point the neighbouring seed is used to sample the neighbouring region of the PES.

Figure S2 shows how our conformational sampling methodology samples the torsional PES for ethane. The three colours utilised correspond to those samples derived from the three energetic minima of ethane. Each minimum energy structure yields a clearly defined band of sampling of the torsional degree of freedom of ethane. Each band possesses a range of torsional sampling in excess of 100°. This range is sufficiently coarse to sample the vast majority of the torsional potential of ethane. We believe Figure S2 to suffice as a proof of concept of our methodology. The thorough sampling of a molecular PES can be accomplished by the normal mode sampling of a series of locally reconstructed PESs.


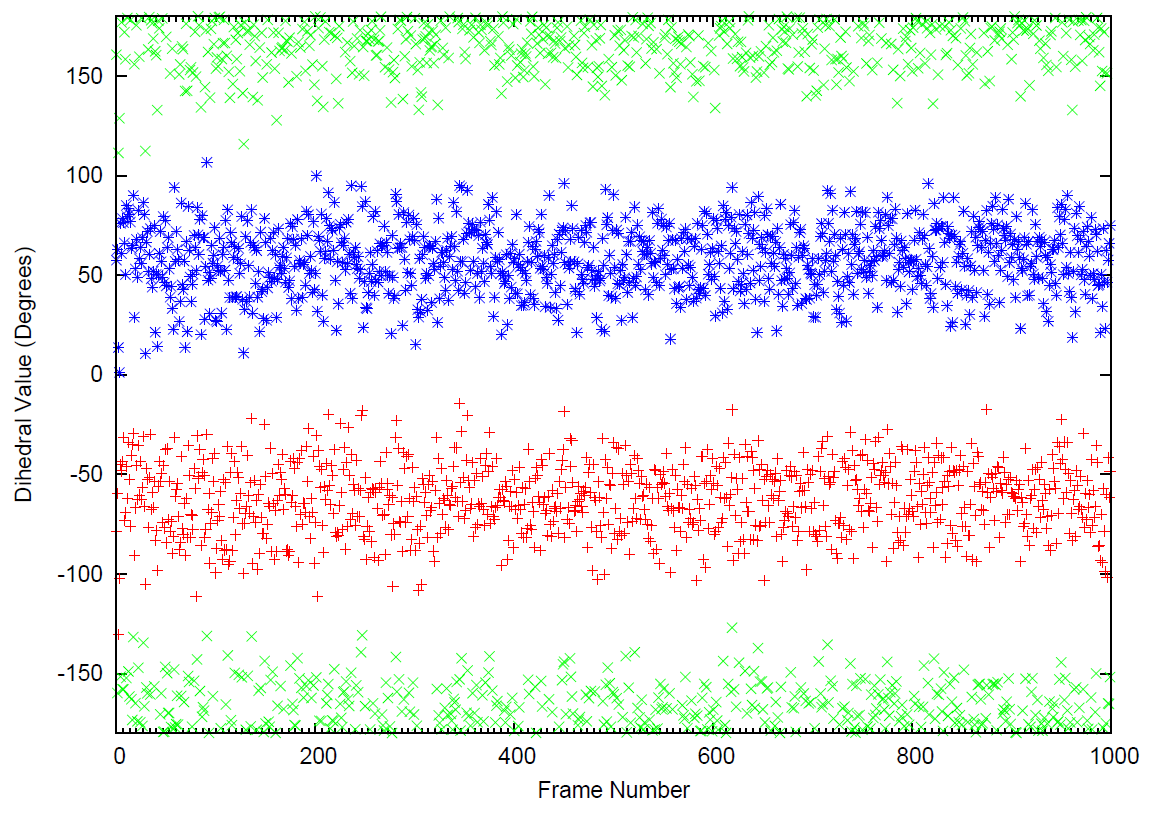


**Figure S2.** Torsional sampling of ethane based on the normal mode conformational sampling methodology presented in Section 2.3. The three colours correspond to samples that have been generated from the three energetic minima of ethane. The individually coloured bands have ranges that exceed 100° of torsional sampling. This range is sufficiently coarse to allow for a thorough exploration of the PES of ethane.

This methodology is, however, not free from pitfalls. For highly flexible systems such as carbohydrates, the sheer number of minima separated by low energy barriers on the PES necessitates a huge number of seeding structures for the normal mode conformational sampling we have proposed. Section 4.3 of the main text illustrates the problems faced by our kriging methodology in undertaking such extensive conformational sampling.

1. The Jacobian $\boldsymbol{J}$ is defined as the derivative of the list of all first-order partial derivatives of a function $\boldsymbol{f}\boldsymbol{:}\mathbb{R}^{\boldsymbol{n}}\boldsymbol{\to}\mathbb{R}^{\boldsymbol{m}}$, with respect to those degrees of freedom, $\boldsymbol{x}$, over which $\boldsymbol{f}$ is defined. Taking the case of $m=1$, we see that $\boldsymbol{J}$ takes the form of $\left[ \partial f/\partial x_{1},\ldots,\partial f/\partial x_{n} \right]^{\top}$, which is the form used here. Of course, this list of (scalar) components is equivalent to the gradient of a scalar field, $\boldsymbol{\nabla}f$, but we prefer to work with its components. [↑](#footnote-ref-1)
